# Genome-wide identification, characterization and expression profiling of gibberellin metabolism genes in jute

**DOI:** 10.1186/s12870-020-02512-2

**Published:** 2020-07-01

**Authors:** Ummay Honi, Md. Ruhul Amin, Shah Md Tamim Kabir, Kazi Khayrul Bashar, Md. Moniruzzaman, Rownak Jahan, Sharmin Jahan, Md. Samiul Haque, Shahidul Islam

**Affiliations:** 1 Basic and Applied Research on Jute Project, Bangladesh Jute Research Institute, Manik Mia Avenue, Dhaka, 1207 Bangladesh; 2 Department of Biotechnology and Genetic Engineering, University of Development Alternative, Dhaka, Bangladesh; 3 Bangladesh Jute Research Institute, Manik Mia Avenue, Dhaka, 1207 Bangladesh

**Keywords:** Jute, Gibberellin, Natural fiber, Biodegradable, Tissue specificity, Feedback regulation

## Abstract

**Background:**

Gibberellin (GA) is one of the most essential phytohormones that modulate plant growth and development. Jute (*Corchorus* sp.) is the second most important source of bast fiber. Our result has shown that exogenous GA can positively regulate jute height and related characteristics which mean increasing endogenous GA production will help to get a jute variety with improved characteristics. However, genes involved in jute GA biosynthesis have not been analyzed precisely.

**Results:**

Genome-wide analysis identified twenty-two candidate genes involved in jute GA biosynthesis pathway. Among them, four genes- *CoCPS, CoKS, CoKO* and *CoKAO* work in early steps. Seven *CoGA20ox*s, three *CoGA3ox*s, and eight *GA2ox*s genes work in the later steps. These genes were characterized through phylogenetic, motif, gene structure, and promoter region analysis along with chromosomal localization. Spatial gene expression analysis revealed that 11 *GA oxidases* were actively related to jute GA production and four of them were marked as key regulators based on their expression level. All the biosynthesis genes both early and later steps showed tissue specificity. *GA oxidase* genes were under feedback regulation whereas early steps genes were not subject to such regulation.

**Conclusion:**

Enriched knowledge about jute GA biosynthesis pathway and genes will help to increase endogenous GA production in jute by changing the expression level of key regulator genes. *CoGA20ox7, CoGA3ox2, CoGA2ox3, and CoGA2ox5* may be the most important genes for GA production.

## Background

Gibberellin (GA), identified by Dr. E. Kurosawa in 1926, is one of the key phytohormones for plant growth and development. This classical phytohormone participates in different physiological processes like seed germination [1, 2], shoot elongation [3], leaf expansion [4], flower development [5], and fruit- senescence [6]. It ultimately induces cell elongation and division; moreover, it accumulates starch [7] and thus has the ability to influence overall plant growth. GAs form a large family of diterpene hormones with 130 members identified in plants, fungi and bacteria [8]. All of them have either 19 or 20 carbon atoms and classified as C19-GAs and C20-GAs respectively. Though plants have hundreds of GAs, only a few of them such as GA_1_ and GA_4_ are bioactive [8].

GA biosynthesis pathway which produces active GAs from geranylgeranyl diphosphate (GGDP, a common precursor of terpenes in plants), is well defined in model plants and most of the genes encoding GA biosynthesis enzymes have been identified [9, 10]. The pathway is distinctly separated into early and later steps [11]. Early steps include conversion of GGDP to GA_12_ whereas later steps include conversion of GA_12_ to GAs. In the first step, GGDP is converted to *ent*-kaurene by two enzymes from terpene synthases (TPSs) family named *ent*-copalyl diphosphate synthase (CPS) and *ent*-kaurene synthase (KS). Then *ent*-kaurene is oxidized to GA_12_ by the activity of another two enzymes named *ent*-kaureneoxidase (KO) and *ent*-kaurenoic acid oxidase (KAO), from cytochrome P450 monooxygenases (P450s). In the later steps, GA20 oxidase and GA3 oxidase catalyze stepwise oxidation to produce various GA intermediates and bioactive GAs like GA_1_, GA_4_ while GA2 oxidase converts them to inactive GAs such as GA_8_, GA_34_ respectively [12].

All the early steps genes are encoded by single or few genes [13]. Though some of the plants have multiple homologous genes, only one of them participates in GA metabolism. For instance, rice has three copies of OsCPS and 11 copies of OsKS-like gene families. However, only OsCPS1 and OsKS1 participate in *ent*-kaurene biosynthesis [14]. On the contrary, GA oxidases are encoded by small gene families [10, 15] and show different expression pattern in different organs [16] and have a distinct functional role. As an example, *MaGA3ox4* has higher expression in young banana fruits while it is lowly expressed in mature fruits. Similarly, the expression level of *MaGA20ox3* is high in leaves, bracts, and young fruits but low in roots, false stems and mature fruits [17]. Moreover, some of the *GA oxidases* maintain GA homeostasis in the plant through feedback regulation [15, 18, 19] and feed forward regulation [20–22]. However, early steps genes i.e. *CPS, KS, KO,* and *KAO* are not subject to feedback control [16, 23] in plants.

Later steps genes are more important in comparison to early steps genes in the regulation of endogenous bioactive GA level [11]. For example- loss of function of *GA20 oxidase* and *GA3 oxidase* caused dwarf phenotype, such as Green Revolution sd-1 [10, 19, 24] whereas no phenotypic alteration was found in the transgenic plants overexpressing *AtCPS and AtKS* [25]. On the other hand, ectopic expression of *GA20ox* resulted into taller plants and larger organs in *Arabidopsis* [26, 27] potato [28], tobacco [29], and maize by increasing endogenous GA levels. Likewise, the dwarf phenotype with dark green leaves was evidenced in transgenic plant over expressing *JcGA2ox6* [30].

Jute, known as golden fiber, is one of the longest and cheapest natural fibers, covering ∼80% of global bast fiber production [31]. It is well known for its durability and versatility. Most importantly, it is biodegradable, renewable and eco-friendly [32]. Hence, jute and jute products are gaining popularity globally and expected to increase by 200% by 2021 [33] which demand more yield. Though both *Corchorus olitorius* and *Corchorus capsularis* are commercially cultivated, the primary source of the fiber is *Corchorus olitorius* since this species is grown in around 80% jute growing areas in the world. The previous studies showed that exogenous GA spray on jute caused the elongated fiber cell along with longer stem and internode [34]. Also, GA induced cell wall thickness and a higher diameter of fiber cell [35]. Moreover, our experiment stated that GA sprays increased internode length of jute which finally created the taller plant than control (Unpublished data). Therefore, GA plays a crucial role in jute growth and development.

GA metabolism pathway is explained in various plant species, such as *Arabidopsis* [13, 15, 16], pumpkin [36], rice [10], pea [37], maize [38], *Salvia miltiorrhiza* [39] and banana [17]. In addition, this knowledge has been extensively used to develop plants having altered GA levels and desired characteristics. For instance– the silencing of *GA2 oxidase* led to transgenic tobacco with increased height and a higher number of xylem cells [40]. Though scientists have already released the genome of two jute species [41], GA metabolism genes in jute are not identified yet. Here, we explored phylogenetic relationship among identified jute GA biosynthesis genes along with their conserve region, gene structure, chromosomal location and duplication event. Moreover, we investigated the effect of exogenous GA spray on jute growth with the GA inhibitor paclobutrazol (PAC) as a contrast. In addition, we checked their expression in different tissues and observe their tissue specificity to identify genes that may trigger GA biosynthesis in jute. This will pave the way to elucidate GA regulation in jute and have jute variety with better agronomic traits.

## Results

### Identification of GA metabolism pathway genes in jute

In order to isolate seven types of GA metabolism genes- *CPS, KS, KO, KAO, GA20 oxidase, GA3 oxidase,* and *GA2 oxidase* in *C. olitorius,* we retrieved respective sequences from *Arabidopsis*, banana, and rice (Additional file 1: Table S1) and used those sequences as the query for BLASTP against jute genome sequences. We have found 22 candidate genes including one *CPS* gene (*CoCPS*), one *KS* gene *(CoKS)*, one *KO* gene (*CoKO*), one *KAO* gene (*CoKAO*), seven *GA20ox* genes (*CoGA20ox1–7*), three *GA3ox* genes (*CoGA3ox1–3*) and eight *GA2ox* genes (*CoGA2ox1–8*).

### Early steps genes

**Fig. 1:**
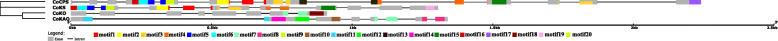
Distribution of conserved motifs and comparative gene structure analysis among early steps genes of jute GA biosynthesis pathway. Twenty conserved motifs were identified for all the four genes using online tool MEME and showed by different colored boxes. Intron exon distributions were done by GSDS tool. Introns and exons are represented by black lines and ash colored boxes respectively

Jute genome has single genes for all the four enzymes involved in early steps. After searching Pfam and SMART database we have found that both CoCPS and CoKS enzymes contain Terpene synthase, N-terminal domain, and Terpene synthase family, metal-binding domain. They were located in chloroplast having molecular weight (Mw) 91.81 kDa and 84.15 kDa respectively. Numbers of introns were relatively higher in these two genes, 14 and 11 respectively (Fig. 1). The result was almost similar to *Arabidopsis*, having 16 introns in both *AtCPS* and *AtKS*. Being a member of cytochrome P450, CoKO and CoKAO have p450 domain. CoKO and CoKAO was located in chloroplast and endoplasmic reticulum respectively. The molecular weight of CoKO was 56.24 kDa while it was 58.19 kDa for CoKAO. Both *CoKO* and *CoKAO* had eight exons and seven introns (Fig. 1). Predicted isoelectric point (pI) for all these four genes varied from 5.55 to 9.15. Detailed information about these genes is given in Table 1.

**Table 1:** Sequence features of GA metabolism genes in *Corchorus olitorius*

Gene name	Accession number	Coding sequence length (bp)	Intron (no.)	Exon (no.)	Isoelectric point (pI)	Molecular weight (kDa)	Sub cellular localization
*CoCPS*	OMP05060.1	797	14	15	5.70	91.81	**Chloroplast**
*CoKS*	OMO77892.1	737	11	12	5.55	84.15	**Chloroplast**
*CoKO*	OMO74862.1	494	7	8	6.45	56.24	**Chloroplast**
*CoKAO*	OMP12279.1	501	7	8	9.15	58.19	**Endoplasmic reticulum**
*CoGA20ox1*	OMP00493.1	388	2	3	5.34	43.80	Cytoplasm
*CoGA20ox2*	OMO97972.1	379	2	3	5.96	43.14	Cytoplasm
*CoGA20ox3*	OMO89803.1	390	2	3	5.75	44.56	Cytoplasm
*CoGA20ox4*	OMO89804.1	383	2	3	5.66	43.61	Cytoplasm
*CoGA20ox5*	OMO89806.1	360	2	3	8.09	41.05	Cytoplasm
*CoGA20ox6*	OMO89807.1	383	2	3	5.85	43.55	Cytoplasm
*CoGA20ox7*	OMO56094.1	383	2	3	6.12	43.40	Cytoplasm
*CoGA3ox1*	OMO80533.1	347	1	2	6.38	39.00	cytoplasm
*CoGA3ox2*	OMO66852.1	368	1	2	7.66	40.78	Cytoplasm
*CoGA3ox3*	OMO57996.1	362	1	2	6.20	40.63	Cytoplasm
*CoGA2ox1*	OMP12162.1	338	2	3	5.79	37.32	Cytoplasm
*CoGA2ox2*	OMP04507.1	305	3	4	6.77	35.12	Cytoplasm
*CoGA2ox3*	OMO92642.1	333	2	3	8.80	37.50	cytoplasm
*CoGA2ox4*	OMO72387.1	359	2	3	7.05	40.53	Cytoplasm
*CoGA2ox5*	OMO65667.1	341	2	3	7.64	37.83	Cytoplasm
*CoGA2ox6*	OMO64910.1	310	2	3	5.03	35.06	Cytoplasm
*CoGA2ox7*	OMO64911.1	332	2	3	5.66	37.99	Cytoplasm
*CoGA2ox8*	OMO58522.1	332	2	3	5.58	36.71	Cytoplasm

### Later steps genes

Three types of enzymes GA20ox, GA3ox and GA2ox are involved in the later steps of GA biosynthesis pathway. All the three groups of enzymes belong to iron-dependent oxidoreductase family and contain two conserved domains [2OG-FeII_Oxy (PF00847) and DIOX_N (PF14226)]. These domains were common in all the sequences except CoGA20ox5, where 2OG-FeII_Oxy domain was missing. Their Mw ranges were from 35.06 kDa to 44.56 kDa with a mean value of 46.00 kDa (Table 1). The lowest isoelectric point was found in CoGA2ox6 (5.03) and the highest in CoGA2ox3 (8.80). All the enzymes were located in the cytoplasm.

**Fig. 2:**
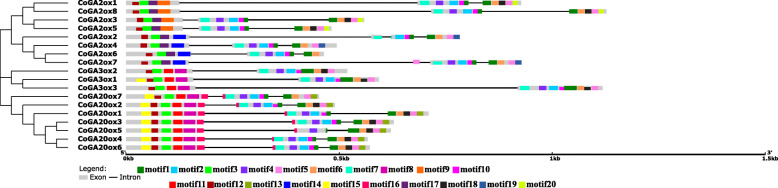
Distribution of conserved motifs and comparative gene structure analysis among jute GA oxidases. Twenty conserved motifs were identified for all the eighteen genes using online tool MEME and showed by different colored boxes. Intron exon distributions were done by GSDS tool. Introns and exons are represented by black lines and ash colored boxes respectively

In gene structure analysis, we found that *GA20 oxidases* genes of jute possess two introns and three exons while *GA3ox* subfamily contained one intron and two exons. Genes from both C-19 GA2ox and C-20 GA2ox subgroups had exons number within 3–4. Some of the members from GAox family-like *CoGA3ox3, CoGA2ox1 and CoGA2ox8* had relatively longer introns (Fig. 2).

### Phylogenetic and conserved motif analysis of GA metabolism genes in jute

#### Early GA biosynthesis genes

**Fig. 3:**
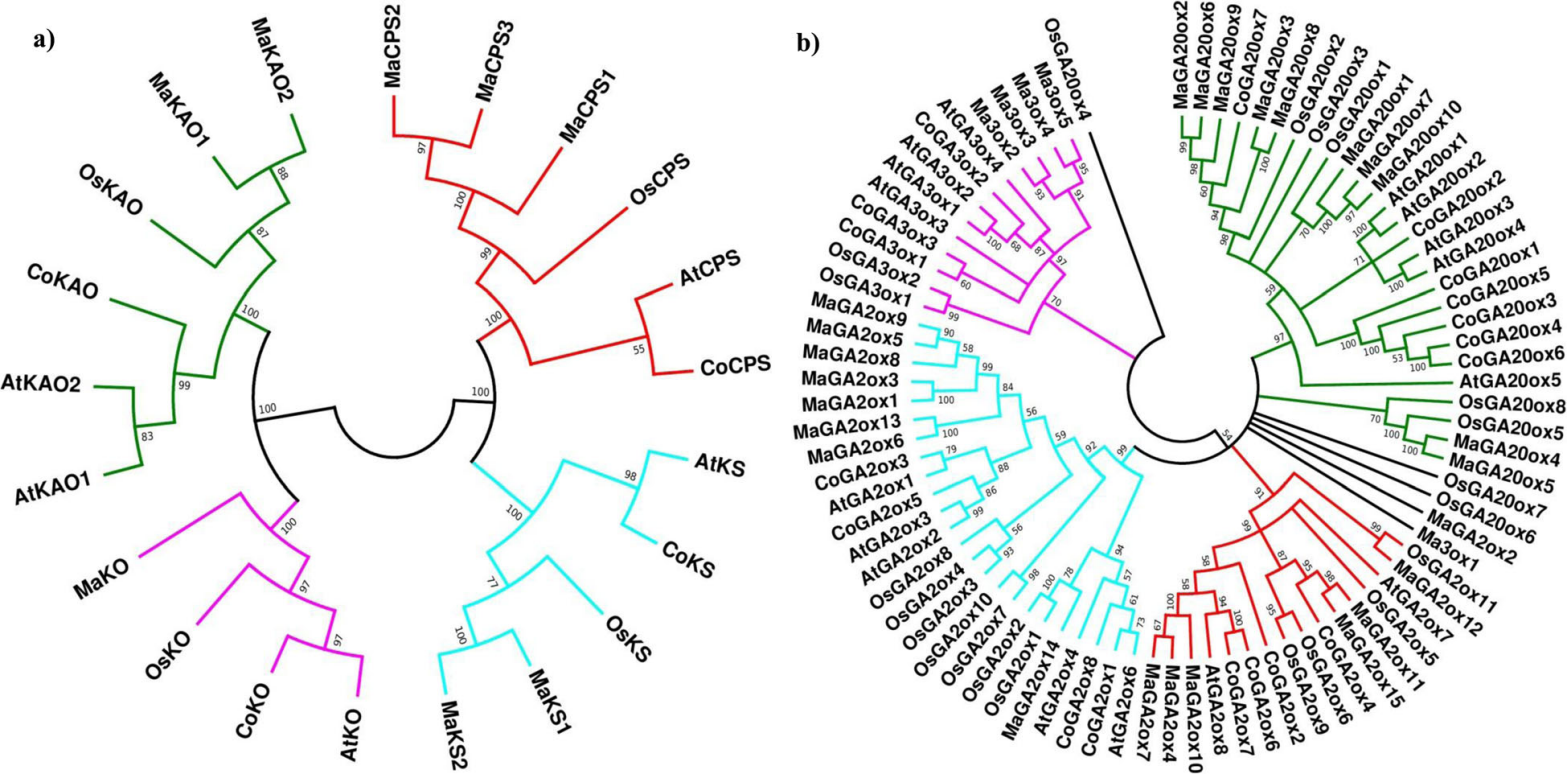
Phylogenetic position of GA metabolism genes. Both the phylogeny tree (**a**) for early GA bio synthesis genes and (**b**) for GA oxidase genes were produced with MEGA 7.0 using the sequences of *Oryza sativa* (Os), *Arabidopsis thaliana* (At) and *Musa acuminata* (Ma) and *Corchorus olitorius* (Co). The accession numbers of protein sequences are mentioned in Additional file 1: Table S1

The phylogenetic analysis revealed that two different protein family, diterpenecyclases (CPS and KS) and Cyt P450 monooxygenase (KO, KAO) were resided in two different branches (Fig. 3a) and four different enzymes remained in four different clades. Moreover, it was demonstrated that early steps enzymes could be separated into monocot and dicot groups.

Motif distribution pattern of these protein sequences showed that both CoCPS and CoKS contained almost similar motifs like 1, 2, 3, 4, 6, 15 and 16 (Fig. 1) since they are from the same protein family. Hence, we speculate that these motifs are specific for diterpene cyclases protein family. However, motif no. 13, 17 and 19 is the distinguishing motif between CoCPS and CoKS where CoCPS contained motif no. 13, 17 and CoKS owned motif no. 19. On the other hand, CoKO and CoKAO proteins shared only one common motif that was motif no. 7. Probably, this was the conserved motif for Cyt P450 monooxygenase protein family. The signature motif for CoKO protein was motif no.18 and for CoKAO these were motif no. 8, 10, 11, 12 and 14. Motif sequences are given in Additional file 1: Table S2.

#### GA oxidases

A neighbor-joining phylogenetic tree was constructed with 55 GA oxidases (GA20ox, GA3ox, GA2ox) sequences from *Arabidopsis*, rice, banana, and jute. The phylogenetic tree was divided into four distinct subgroups- GA20 oxidases, GA3 oxidases, C-19 GA2 oxidases and C-20 GA2 oxidases (Fig. 3b). GA20ox and GA2ox had comparatively larger group than GA3ox. For example- *Arabidopsis*, rice, banana, and jute had 5, 8, 10, and 7 copies of GA20ox respectively whereas they had 4, 2, 4, and 3 copies of GA3ox respectively.

**Fig. 4:**
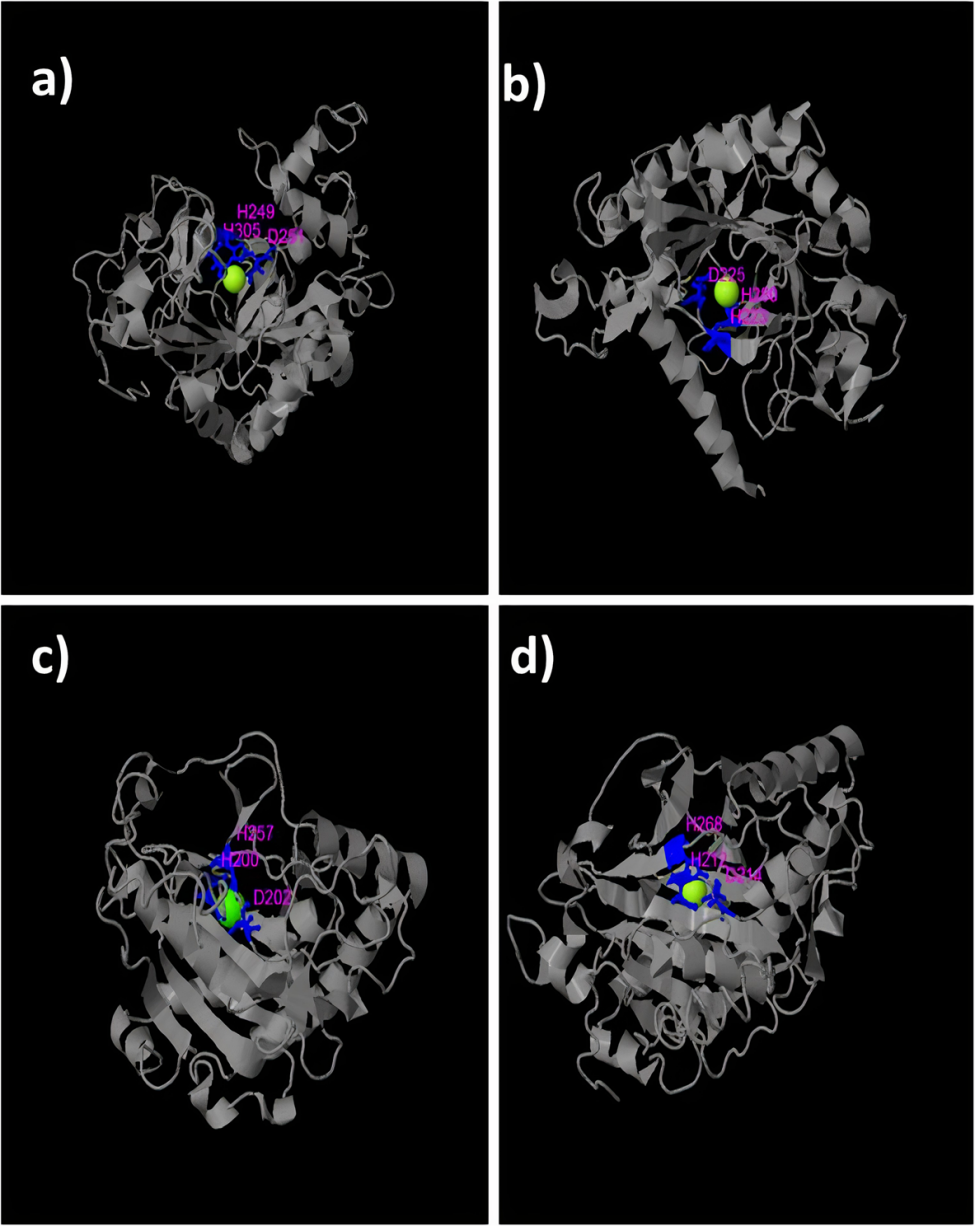
3D structures of jute GA oxidases showing Fe2+ binding motif. The structure of (**a**) CoGA20ox1- representative of GA20 oxidases, (**b**) CoGA3ox1- representative of GA3 oxidases, (**c**) CoGA2ox8- representative of C-19 GA2 oxidases, (**d**) CoGA2ox4- representative of C-20 GA2 oxidases were modeled by I-TASSER server

GA oxidases are from Isopenicillin N synthase family which contains a Fe^2+^-binding motif, an HXD dyad near the amino terminus and a histidine towards the carboxyl terminus [42]. Multiple sequence alignments and 3D structure exhibited that the position of these amino acids were His-249, Asp-251, and His-305 for jute GA20 oxidases (Fig. 4a; Additional file 2: Figure S1) which were His-223, Asp-225 and His-280 for GA3 oxidases (Fig. 4b, Additional file 2: Figure S2). As a member of Isopenicillin N synthase, GA2 oxidases also carry the same motif. Positions of the two His and one Asp residues were at 200, 257 and 202 respectively for C-19 GA2ox (Fig. 4c, Additional file 2: Figure S3). Similarly, C-20 GA2ox contained the HXD dyad at 214 and 217 positions and the second His at 268 (Fig. 4d, Additional file 2: Figure S4). Besides, GA20 oxidases possessed two binding site, NYYPXCXXP sequence at 232–240 position and LPWKET at 151–156 position. However, only two sequences of jute, CoGA20ox2 and CoGA20ox7 had the exact binding site LPWKET while rest five possessed a little bit different sequences, for example- CoGA20ox1, CoGA20ox4, and CoGA20ox6 had M, F, and F respectively instead of L in the first position (Additional file 2: Figure S1).

Jute GA oxidases shared some common motifs like motif no. 1, 3, 6, 12 (Fig. 2). Motif no. 4 was also common except in CoGA2ox2. Similarly, motif no. 2, 7 and 10 were available in all the 17 sequences excluding in CoGA20ox5. Signature motifs for GA20 oxidases were motif no. 13 and 16 while such special motif was not found for GA3 oxidases. As both GA20ox and GA3ox had motif no. 8 and 11, they were probably the function leading motif for these sub families. Unique motifs for C-19 GA2ox and C-20 GA2ox proteins were motif no. 9 and 14 respectively. Both C-19 GA2ox and C-20 GA2ox contained motif no. 17, which was absent in GA20 oxidases and GA3 oxidases. So, this exclusive motif may account for the function difference of GA2ox gene family. Motif sequences are given in Additional file 1: Table S3. Though motif distribution pattern was different in different subgroups, it was very similar within the subgroup of different species which indicates that members from the same subfamily have similar gene structures.

### Finding the *cis*-acting element in the promoters of GA biosynthetic genes

*Cis*-acting elements play a vital role in gene transcription by facilitating the binding of transcription factors. To elucidate the regulatory mechanism of GA metabolism genes, 1000 bp upstream sequences from each transcription start site were searched for *cis*-elements. Available *cis*-regulatory elements were classified in four categories-(a) plant hormone regulation, (b) biotic and abiotic stress, (c) plant growth and development and (d) promoter function (Additional file 2: Figure S5). Stress (biotic and abiotic) responsive *cis*-elements were the maximum in jute GA biosynthesis genes and out of those light-responsive elements (ATC motif, G-box, GT- motif, TCT- motif, 3-AF1 binding site, Box 4, TCCC-motif, ATCT-motif, AE-box, CAG motif, MRE, I-box, GATA-motif, Box ii, CAG-motif, GA-motif, LAMP-element, chs-CMA2a) were the highest. The number of motifs involved in hormonal regulation was the second largest group after stress-responsive motifs in jute GA metabolism genes. Moreover, they contained motifs related to different phytohormone like Salicylic acid (TCA-element, SARE), Auxin (TGA element), Abscisic acid (ABRE), Gibberellic acid (TATC-box, p-box, GARE-motif). Interestingly, out of eighteen GA oxidases only seven genes- *CoGA20ox2, CoGA20ox7, CoGA3ox2, CoGA2ox3, CoGA2ox5, CoGA2ox6* and *CoGA2ox7* contained the motif p-box (Additional file 2: Figure S5). However, jute GA biosynthesis genes had the least number of *cis*-acting elements for plant growth and development (CAT-box, O2-site, GCN4_motif). Available *cis*-regulatory elements in GA promoter region and their description are given in Additional file 1: Table S4.

### Gene ontology annotation (GO)

**Fig. 5:**
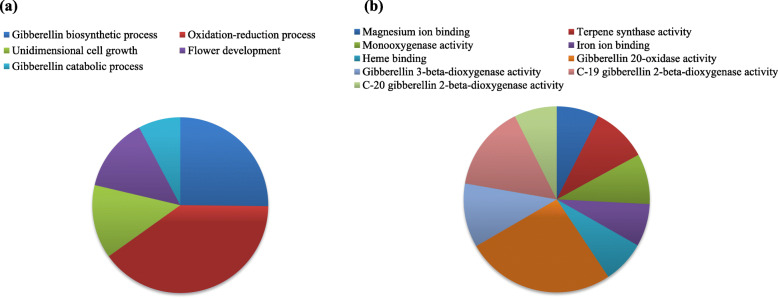
Gene ontology annotation of GA bio synthesis genes. **a** biological process and (**b**) molecular function

The GO analysis implied that most of the jute GA biosynthesis genes involved in the oxidation-reduction processes. They also participated in flower development and unidimensional cell growth (Fig. 5a). These genes also had a wide range of molecular functions like gibberellin 20-oxidase, gibberellin 3-beta-dioxygenase, C-19 gibberellin 2-beta-dioxygenase, C-20 gibberellin 2-beta-dioxygenase activity. In addition, they also had magnesium, iron, and heme-binding capacity (Fig. 5b).

### Chromosomal localization and duplication analysis

**Fig. 6:**
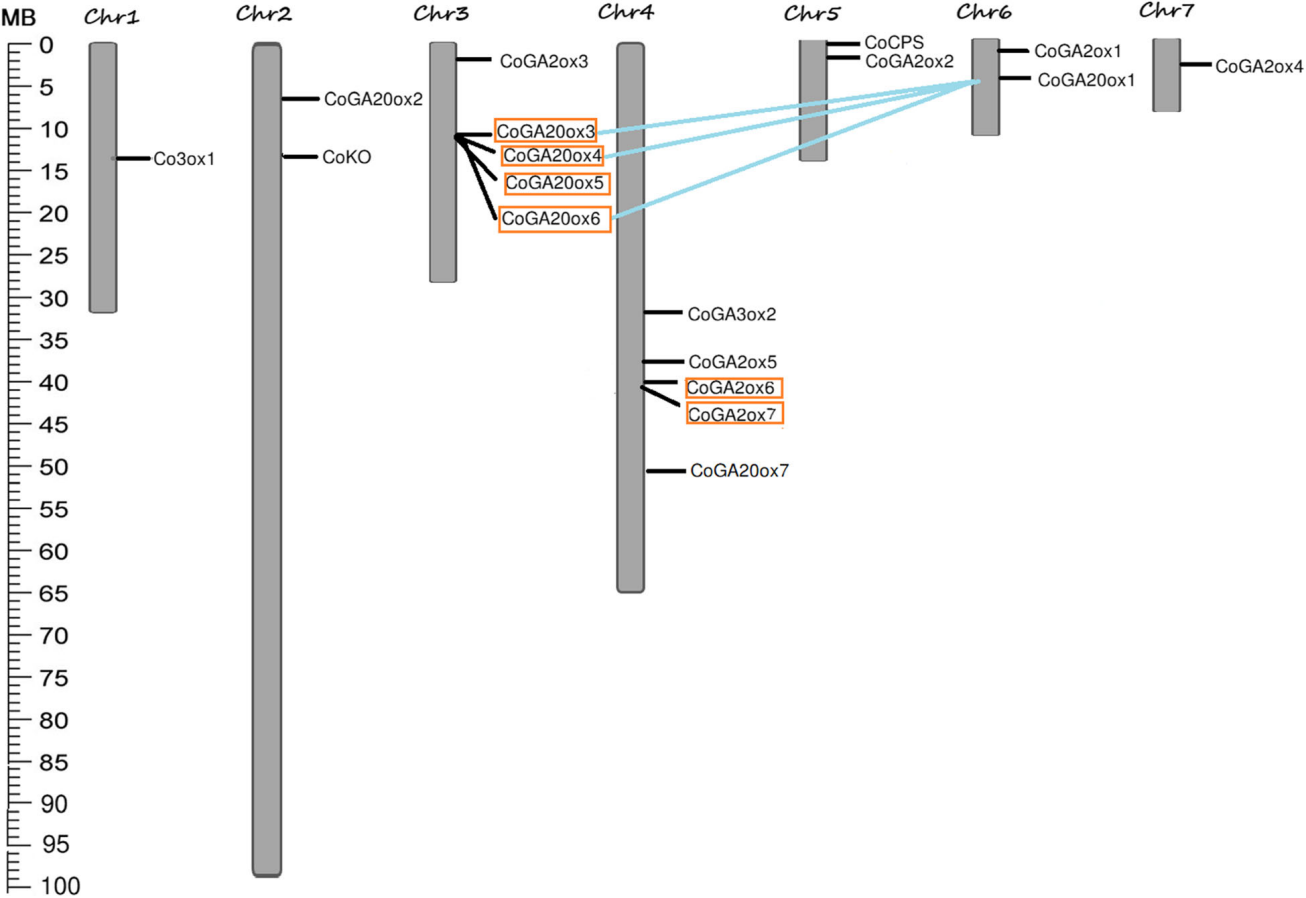
Chromosomal location and duplicated gene pairs of jute GA biosynthesis genes. Eighteen genes were distributed in seven chromosomes of jute. Segmental duplications are indicated by blue line and tandem duplications are marked by orange rectangle. The unit of the ruler is megabases (MB)

To know the distribution pattern of GA biosynthesis genes in different chromosomes, we mapped them on the seven chromosomes of jute and found that most of the genes were located on chromosome 3 and chromosome 4 (Fig. 6). Four genes including *CoKS, CoKAO, CoGA3ox3*, and *CoGA2ox8* were not mapped to any of the jute chromosomes, since, draft assemble of jute only anchored ~ 60% of the total genome.

Duplication is an important mechanism in the process of evolution. Two gene clusters of tandem duplication and three gene pair of segmental duplication were found in jute GA biosynthesis genes (Fig. 6). Ka/Ks ratio was calculated to explore the trend of gene divergence after duplication. Ninety percent of jute GA biosynthesis genes had undergone through purifying selection which means functional divergence might occur before duplication events (Table 2). The time of the duplication event was calculated using synonymous substitution rate. The result revealed that divergence time for segmental duplication was 8.85 to 10.25 million years ago (MYA) and it was from 1.32 to 9.16 MYA for tandem duplicated genes (Table 2).

**Table 2:** Ka/Ks estimation for the duplicated gene pairs

Duplicated gene 1	Duplicated gene 2	Ka	Ks	Ka/Ks	Purifying selection	Duplicate type	Age (MYA)
*CoGA20ox1*	*CoGA20ox3*	0.1569	0.2655	0.5910	Yes	segmental	8.85
*CoGA20ox1*	*CoGA20ox4*	0.1621	0.3047	0.5320	Yes	segmental	10.16
*CoGA20ox1*	*CoGA20ox6*	0.1597	0.3076	0.5192	Yes	segmental	10.25
*CoGA20ox3*	*CoGA20ox4*	0.0651	0.0898	0.7250	Yes	tandem	3.00
*CoGA20ox3*	*CoGA20ox5*	0.1043	0.1494	0.6981	Yes	tandem	4.98
*CoGA20ox3*	*CoGA20ox6*	0.0728	0.0710	1.0261	No	tandem	2.36
*CoGA20ox4*	*CoGA20ox5*	0.1449	0.1844	0.7858	Yes	tandem	6.14
*CoGA20ox4*	*CoGA20ox6*	0.0147	0.0398	0.3689	Yes	tandem	1.32
*CoGA20ox5*	*CoGA20ox6*	0.1460	0.1803	0.8097	Yes	tandem	6.01
*CoGA2ox6*	*CoGA2ox7*	0.0837	0.2748	0.3047	Yes	tandem	9.16

### Spatial expression analysis

**Fig. 7:**
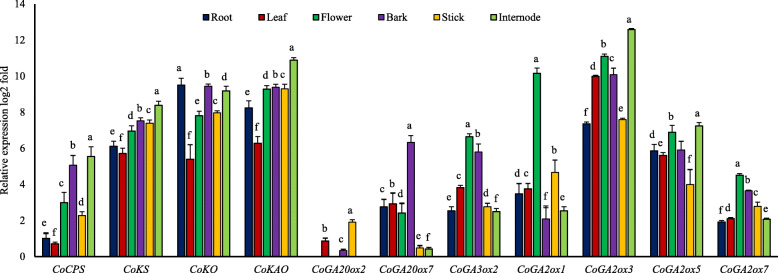
Spatial expression profiles in jute. The qRT-PCR was performed with the RNA isolated from root, leaf, flower, bark, stick, top internode. Relative expression value was determined by 2^-ΔΔC^T method. Normalization was done against *PP2Ac* gene. Expression level of *CoCPS* in the root, was set to 1. The CT value of each gene was the average of three biological and three technical replicates. Columns at each observation followed by the different letter are significantly different according to LSD test at *P* ≤ 0.05. Vertical bars indicate standard errors

After identifying different genes of jute GA biosynthesis pathway we checked their expression in root, leaf, flower, stick, bark and top internode to know whether they had organ-specific expression pattern. In general, the early steps genes showed ubiquitous expression in all the organs analyzed. *CoKAO* had higher expression compared to other three genes. *CoCPS, CoKS* and *CoKAO* had similar expression pattern having the highest expression in top internode and the lowest expression in leaf. The expression of *CoKO* gene was higher in root, bark and internodes while it has its lower expression in leaf (Fig. 7). Notably, most of the genes (*CoCPS, CoKO, CoKAO*) had their highest expression in top internode in comparison to other tissues.

Interestingly, most of the later steps genes (11 genes) were inactive in our considered samples. Only seven of them *CoGA20ox2, CoGA20ox7, CoGA3ox2, CoGA2ox1, CoGA2ox3, CoGA2ox5*, and *CoGA2ox7* had expression in different tissues. To justify our result, we investigated the expression of GA biosynthesis genes by using the RNA-seq data published by Islam et al. [41]. It was very similar to our qRT-PCR data (Additional file 2: Figure S6). Moreover, we also performed PCR analysis with cDNA of these unexpressed genes using qRT-PCR primers (Additional file 1: Table S5) and no band was observed in the gel (data not shown).

For GA20ox family, *CoGA20ox7* was expressed broadly in all the tissues examined while *CoGA20ox2* showed tissue specificity having expression only in leaf, bark, and stick. The expression of *CoGA20ox7* was predominant in bark followed by leaf and root. Overall, *CoGA20ox2* had lower expression than *CoGA20ox7* in almost all the tested parts. *CoGA3ox2*, the only active gene of GA3 oxidase family, exhibited its highest expression in flower and the lowest expression in the top internode (Fig. 7). Among four GA2 oxidases, *CoGA2ox3* appeared to have the highest expression and it was mostly expressed in top internode. Overall, *CoGA2ox5* had a moderate expression having higher expression in internode and flower and less in stick. Though expression level of *CoGA2ox1* was high in flower, it was actually a weekly expressed gene. The *CoGA2ox7* had the least expression in all the considered tissue in comparison to other *GA2 oxidases* (Fig. 7). On the basis of expression pattern, it may be hypothesized that *CoGA20ox7, CoGA3ox2*, *CoGA2ox3* and *CoGA2ox5* were the key regulators for GA production in jute and *CoGA20ox2, CoGA2ox1, CoGA2ox7* were the least important ones.

### Morphological effect of exogenous treatments

**Fig. 8:**
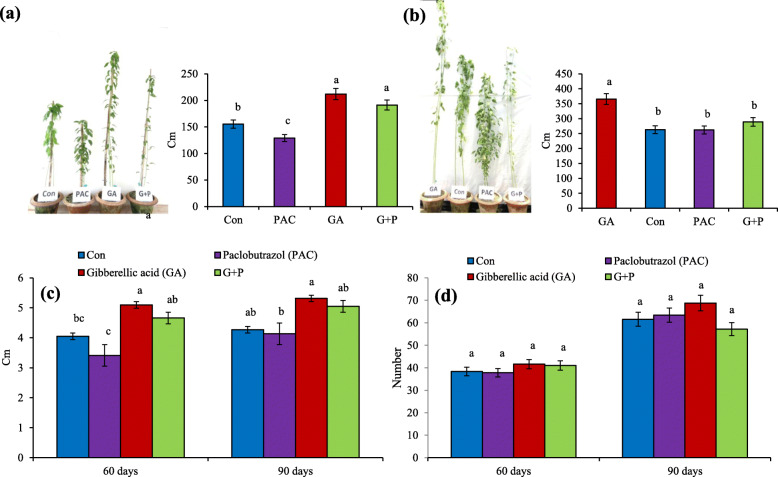
Effect of exogenous treatment on jute morphology. **a** Plant height for 60 days old plant, (**b**) Plant height for 90 days old plant, (**c**) Internode length and (**d**) Node number upon GA, PAC and G + P treatment. Spray was continued every 10 days interval from 30 days to 90 days of age. Columns at each observation time point followed by the same letter are not significantly different according to LSD test at *P* ≤ 0.05. Vertical bars indicate standard errors

Plants were treated with exogenous GA, PAC and G + P solutions to confirm GA’s effect on the jute plant. The results suggested that plant height was significantly increased in GA treated plant (Fig. 8a and b) than the controls. On the other hand, the reverse scenario was found in the case of PAC treatment. In G + P treatment, the plants had moderate height, lower than GA treated plants and higher than PAC treated plants. A similar trend was also found in the case of internode length and node number (Fig. 8c and d). GA increased node number and internode length while PAC caused lower number of nodes and shorter internode in comparison to control. These results indicated that GA has a significant role in plant development.

### Response of GA biosynthesis genes to exogenous treatments

**Fig. 9:**
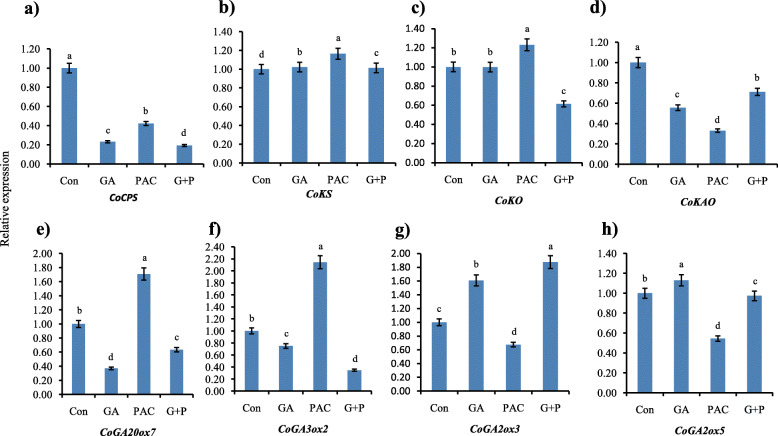
Effects of GA, PAC and G + P on the expression of genes involved in GA biosynthesis. Fold changes of early steps genes (**a**-**d**), *CoGA20ox7* (**e**), *CoGA3ox2* (**f**), *CoGA2 oxidases* (**g**-**h**). The expression was analyzed by using quantitative RT-PCR method and relative expression value was determined by 2^-ΔΔC^T method. Normalization was done against *GAPDH* gene. Expression level of tissue treated with water considered as control. The CT value of each gene was the average of three biological and three technical replicates. Columns at each observation followed by the different letter are significantly different according to LSD test at *P* ≤ 0.05. Vertical bars indicate standard errors

Thirty days old jute plants were treated with distilled water (con), GA, PAC, and G + P in order to know how GA metabolism pathway genes react to exogenous spray. In this experiment, only the top internode was collected as RNA sample for qRT-PCR analysis. *CoCPS* and *CoKAO* were down-regulated while *CoKS* was a little bit up-regulated in comparison with control in all the treatments. *CoKO* remained unchanged in GA treated samples whereas it was up-regulated in PAC treatment and down regulated in G + P treatment (Fig. 9a-d).

Based on spatial expression analysis, *CoGA20ox7, CoGA3ox2, CoGA2ox3*, and *CoGA2ox5* were selected to check their response to GA, PAC, and G + P treatments. *CoGA20ox7* was down-regulated upon GA and G + P, 0.63 times and 1.07 times respectively whereas they were significantly up-regulated upon PAC treatment, 1.3 times compare to the control. The expression level of *CoGA3ox2* also followed the same trend having 0.25 and 1.7 times down-regulation and 1.3 times up-regulation than the control after GA, G + P and PAC treatment respectively (Fig. 9e-f). On the contrary, GA and G + P spray enhanced the expression level of *CoGA2ox3, CoGA2ox5* while PAC spray decreased their expression level (Fig. 9g-h). Overall, the expression changes were lower in GA treatments than in PAC and G + P.

## Discussion

### Gene identification, phylogenetic relationship, motif analysis, and gene structure

#### Early steps enzymes

Enzymes involved in the early stages of GA metabolism pathway in jute are encoded by single genes. This finding is fully consistent with previous study where it was concluded that early steps of GA metabolism pathway genes are encoded by single or few genes [13]. For example- *Arabidopsis* has one copy of *CPS, KS*, and *KO* and two copies of *KAO* genes [43]. Finding from phylogenetic relationship and gene structure analysis reveals jute has more close relation with *Arabidopsis* than rice and banana (Fig. 3a) which justify that early step enzymes could be divided into monocot and dicot [17]. Subcellular localization by Plant-mPLoc showed that CoCPS, CoKS, and CoKO were located in chloroplast and CoKAO was located in the endoplasmic reticulum. This result is very much harmonious with previous studies. For example - AtCPS and AtKS1 are localized in chloroplast while AtKO is remained in the outer envelope of chloroplast and AtKAO1, AtKAO2 are destined in the endoplasmic reticulum [44].

#### Later steps enzymes

We have identified 18 candidate genes for three types of GA oxidases in jute. Among them, seven GA20 oxidases, three GA3 oxidases, and eight GA2 oxidases are available in jute while *Arabidopsis* possess five GA20 oxidases, four GA3 oxidases, and seven GA2 oxidases. Rice genome has 8, 2 and 11 copies of GA20ox, GA30x and GA2ox respectively. So, GA20ox and GA2ox have a larger number of genes which proves a more dynamic evolutionary route of these two groups. This also causes relaxed selective pressure or loosened constraints in the process of evolution. On the other hand, GA3ox has fewer numbers of genes that means the family is more conserved than GA20ox and GA2ox [17].

The phylogenetic analysis of GA oxidases showed that they are segregated into four distinct subgroups (GA20ox, GA3ox, C-19 GA2ox, and C-20 GA2ox). This result is quite similar to *Arabidopsis*, rice, maize and soybean [17, 38, 45]. Identification of unique motifs for all the subgroups further justified the classification. In each subgroup, it was observed that GA oxidases from the same species were more closely related than GA oxidases from other species which ultimately meant that GA oxidase expansion occurred early in the evolution of this protein family.

Though the catalytic domain was well conserved within GAox family proteins, there was variation in gene structure in different subfamilies within jute. More similarity in gene structure with more closely related members of the subfamily suggests selective diversification of the GAox family. The majority of the GAox subfamilies in jute contained three exons. This also emphasizes on selective diversification. Some members of the jute GAox family had relatively longer introns. These introns may have been evolved from unequal crossing over [45].

### Morphological effect of exogenous treatment

GA treated jute plants were relatively tall whereas PAC treated plants were relatively dwarf. This finding is similar to other fiber crops like hemp [46] and flax [47]. Similarly, GA caused increased node number and internode length. These observations proved that GA influences vegetative stem growth by increasing both the number and length of the internodes. GA causes increased cell elongation and division by inducing transcription of genes involved in these processes. As an example- in the elongating stem of rice and *Arabidopsis*, some of the genes encoding xyloglucan endotransglycosylases (XETs) and expansins have been up-regulated by GA [48–50]. XET is responsible for plasticity of the cell wall while expansins are involved in plant cell wall loosening. Some of the genes encoding for cyclin-dependent protein kinases also showed ectopic expression in intercalary meristem in rice upon GA treatment [51]. However, in G + P treatment, plant height was moderate justifying that PAC inhibited plant growth mainly by decreasing GA biosynthesis and it was partially rescued by GA.

### Tissue specificity of GA metabolism genes and their response to exogenous treatment

Jute GA biosynthesis genes showed obvious tissue specificity, similar to many other plants [17, 39, 43]. However, most of the *GA oxidases* genes of jute did not participate in GA biosynthesis. For example, among five *GA20 oxidases* of jute only two (*CoGA20ox2* and *CoGA20ox7*) were expressed. This result ultimately proves the redundant functionality of GA oxidases [16]. Interestingly, promoter analysis of these genes showed that most of the expressed genes contain a specific *cis*-acting element that is p-box (Additional file 2: Figure S5). Therefore, p-box might have important role in the regulation of *GA oxidases*. It was also noticeable that top internode was the mostly active tissue where most of the genes of GA metabolism pathway had their highest expression. These results indicate the regulation of GA biosynthesis at the tissue and organ level and also reveals that the localization of *CoGA2ox3* and *CoGA2ox5* expression within an organ affects both GA homeostasis and GA levels, and thereby growth. Generally, growing organs like developing leaves, expanding internodes contain the highest amount of bioactive GA [52] and these organs have a high expression level of GA-biosynthetic genes [53]. Consequently, for checking the effect of exogenous GA, PAC, and G + P treatments, samples were collected only from top internode. In this experiment, *CoGA20ox7*, *CoGA3ox2* were down-regulated in GA treatment and showed reverse expression in PAC treated samples. The plant has its own homeostasis system to maintain optimum GA level to continue its natural growth and development through a mechanism named feedback regulation [54]. When plants were treated with exogenous GA, the quantity of endogenous GA was increased and *CoGA20ox7*, *CoGA3ox2* decreased their expression to lower the synthesis of GA. Likewise, plants treated with PAC had a lower amount of endogenous GA since PAC inhibits GA synthesis. As a result, *CoGA20ox7, CoGA3ox2* increased their expression to minimize the effect of PAC. On the other hand, *CoGA2ox3, CoGA2ox5* which were responsible for inactivating active GA, showed just reverse expression pattern. This was due to another homeostasis mechanism named feed forward regulation of GA2ox genes [20–22]. Overall, both in up-regulation and down-regulation GA had weaker effect in gene expression than PAC which ultimately means that in control condition jute had optimum level of GA in top internode. According to Acheampong et al. [55] when a specific organ has minimal amount of GA for its growth, it shows high response to PAC and minimal changes to GA. For G + P treatment, plants were firstly treated with PAC that means they had a low amount of GA. However, after 2 days when they were treated with GA, the effect of PAC was minimized. Therefore, in these samples, the gene expression change was similar but higher than GA treated samples. For early steps genes, we did not get any feedback regulation. Indeed this is already proven that early steps genes are not under feedback regulation [23]. However, these genes showed altered transcript level upon GA, PAC and G + P treatment. This phenomenon is difficult to explain as two of them CoCPS and CoKS interact with (E,E)-4,8,12-trimethyltrideca-1,3,7,11-tetraene (TMTT) biosynthesis pathway [56]. Moreover, PAC competes with KO for inhibiting GA biosynthesis [57]. Therefore, the reasons for this phenomenon need to be experimentally evidenced.

Though jute *GA oxidases* genes changed their expression to maintain GA homeostasis in jute still we got different morphology like increased plant height, internode length upon continuous GA spray which was also a common phenomenon for carrot [58] and apple [59]. Actually treating jute with GA and PAC in every 10 days altered endogenous GA level of jute and alter hormone homeostasis, thus influence plant growth.

## Conclusion

In this study, we have identified 22 candidate genes for seven enzymes involved in GA metabolism in jute and classified them into seven gene families including CoCPS, CoKS, CoKO, CoKAO, CoGA20ox, CoGA3ox, and CoGA2ox. Phylogenetic analysis, motif distribution pattern and gene structure study proved that GA metabolism genes in jute maintain conservation and divergence. Moreover, through spatial expression analysis, we have proved their tissue specificity and found top internode as the most active zone where most of the GA metabolism genes have comparatively higher expression. We also found that only later steps genes are feedback regulated. Combined these results we have finally identified four key regulatory genes. This comprehensive analysis of GA biosynthesis genes will be a foundation for the genetic improvement of jute and other closely related plants with agricultural importance.

## Methods

### Sequence acquisition, alignment, and phylogenetic tree construction

All the sequences involved in GA biosynthesis were retrieved from TAIR (The *Arabidopsis* Information Resource, http://www.Arabidopsis.org), Rice Genome Annotation Project Database (http://www.rice.plantbiology.msu.edu/), The Banana Genome Hub (http://banana-genome.cirad.fr/blast) and the NCBI (http://www.ncbi.nlm.nih.gov/). To identify homologs of these genes in jute, BLAST search (BLASTP) was carried out against *Corchorus olitorius* cultivar: O-4 genome (https://www.ncbi.nlm.nih.gov/bioproject/215141). Sequences were accepted from BLAST which had an expected threshold lower than 1e-30. All the sequences are listed in Additional file 1: Table S1. Expasy ProtParam tool (http://web.expasy.org/protparam/) was used to determine physical and chemical properties like the molecular weight, theoretical pI and so on. Subcellular localization was investigated through Plant-mPLoc (http://www.csbio.sjtu.edu.cn/bioinf/plant-multi/). Multiple sequence alignments of the *Arabidopsis*, rice, banana, and jute were conducted by Clustal Omega tool (http://www.ebi.ac.uk/Tools/msa/clustalo/). An unrooted phylogenetic tree was generated by using the neighbour-joining (NJ) method with poisson correction, pairwise deletion and 1000 bootstrap replicates parameters by MEGA version 7 [60].

### Domain, motif, gene structure, and 3D structure determination

The protein sequences of predicted jute GA metabolism genes were subjected to Pfam (http://pfam.sanger.ac.uk/search) and SMART (http://smart.emblheidelberg.de/) database to identify their conserved domain. To obtain their characteristic motif, Multiple Expectation Maximization for Motif Elicitation (MEME) (http://meme-suite.org/) was employed with the parameters as follows- maximum numbers of motifs was set to 20, the optimum motif width was 6–50 amino acid, and 0 or 1 single motif in each sequence of the model. The phylogenetic trees together with annotation file in Generic Feature Format Version 3 (GFF3) were prepared for visualization of the Gene Structure Display Server (GSDS) 2.0 (http://gsds.cbi.pku.edu.cn/) to determine the intron-exon structure. Phylogenetic tree, conserved motif and gene structures were integrated by using a Scalable Vector Graphics (SVG) editor Inkscape 0.92.4 (https://inkscape.org/). The three dimensional (3D) structure with Fe^+ 2^ binding site was predicted through an online tool named I-TASSER (Iterative Threading Assembly Refinement) (http://zhanglab.ccmb.med.umich.edu/I-TASSER/).

### Promoter analysis and gene ontology annotation

For promoter analysis, 1000 bp upstream from the transcription start site was retrieved from the *Corchorus olitorius* genome by using custom Perl scripts. Those putative *cis*-acting elements were investigated through an online promoter analysis tool named PlantCare (http://bioinformatics.psb.ugent.be/webtools/plantcare/html/). Gene ontology analysis was performed with Blast2GO software (https://www.blast2go.com/). The GO terms for each of the two main categories- biological process and molecular function were obtained from sequence similarity with the default parameters.

### Chromosomal localization and duplication analysis

Distribution of the GA biosynthesis genes in the different chromosome of jute was represented with a schematic diagram. Generally, proteins that had more than 70% identity and separated by no more than five other genes within 100 kb region were considered as tandem duplications [61]. Similarly, genes separated by more than five genes were considered as segmental duplication.

### Determination of ka/ks ration

Amino acid sequences of duplicated gene pairs were aligned by using Clustal Omega program. Ka (the number of nonsynonymous substitutions per nonsynonymous site) and Ks (the number of synonymous substitutions per synonymous site) ratio was calculated by PAL2NAL (http://www.bork.embl.de/pal2nal/) through the codeml program in PAML. In general, Ka/Ks = 1 means neutral selection; Ka/Ks < 1 means purifying selection; Ka/Ks > 1 means accelerated evolution with positive selection [62]. The divergence time ‘t’ was estimated with the equation, “t = Ks/2r” where r = 1.5 × 10^− 8^ according to Islam et al. [41].

### Plant materials

Tossa jute (*C. olitorius* cv. O-4) was used in this experiment for both morphological and molecular analysis. Seeds were collected from Gene Bank of Bangladesh Jute Research Institute and its accession number was 1808. They were grown in the greenhouse of BARJ under 16 h light and 8 h dark at 32 °C in 36 earthen pots (30 × 30 cm) and each of the pot contained four seeds. The earthen pots were prepared with a mixture of vermiculite and organic soil (1:1). Among them, 12 pots were used for morphological data collection, 12 pots were used for spatial expression analysis and the remaining were used for checking the effect of GA, PAC and G + P spray. The pots were maintained in the greenhouse in a control environment till the end of the experiments.

### GA, PAC, G + P treatment

GA (LOBA Chemie) and Paclobutrazol (PAC)- the inhibitor of GA (Sigma-Aldrich, Germany) solution were prepared at 100 ppm with distilled water and 0.1% tween 20 (wetting agent). In the case of G + P spray, plants were sprayed with PAC before 2 days of GA spray and this combined treatment (G + P) was used to verify the relation between the effect of GA and PAC.

### Morphological data collection

At the age of 30 days, plants were sprayed individually with tween 20 formulated GA, PAC, and G + P solution at 100 ppm concentration by using a low-pressure hand-wand sprayer. During spray, 60% humidity and 25–27 °C temperature were maintained. Control plants were treated with distilled water containing 0.1% Tween 20. The spray was continued at 10 days interval from 30 days to 90 days of plant age. Plant height of all treated plants along with the control was measured by a meter scale at 60 days aged (4th dose of spray) and 90 days aged plants (7th dose of spray). Internode length was determined by dividing the total length of the plant with a total node number.

### RNA sample collection

For conducting spatial gene expression analysis, root, leaf (5th leaf), bark and stick (9 cm from top), top internode (rosette to 5th leaf- around 5.5 cm) samples were collected from 40 days old plants. To check the effect of exogenous spray, top internode (rosette to 5th leaf) was collected as RNA sample after 12 h of spray with 100 ppm of GA, PAC and G + P. All the sampled organs were immediately frozen at liquid nitrogen in the greenhouse and preserved at − 80 °C.

### RNA isolation and cDNA preparation

For RNA isolation one gram of each sample was crushed in liquid nitrogen to a fine powder through a mortar and pestle. Finally, RNA was extracted by the modified CTAB method mentioned in Ahmed et al. [63]. RNA quality was checked by using 1% gel electrophoresis followed by quantification with NanoDrop 2000 spectrophotometer (NanoDrop, ThermoFisher Scientific, USA). All the RNA samples were treated with amplification grade DNase I (Sigma-Aldrich, Germany) to avoid DNA contamination. After that, this pure RNA was used to prepare first-strand complementary DNA (cDNA) using the RevertAid First Strand cDNA Synthesis Kit (Thermo Fisher Scientific, USA) according to the manufacturer’s instructions. Subsequently, the samples were incubated with RNase H (Thermo Fisher Scientific, USA) to degrade the RNA strand of any RNA–DNA hybrids according to the manufacturer’s instructions and preserved at − 20 °C until use.

### Primer design and reverse transcription quantitative real-time PCR (qRT-PCR)

Specific qRT-PCR primers were designed by the online tool IDT (https://sg.idtdna.com/pages/tools/primerquest) and Genscript (www.genscript.com) for all the genes involved in GA biosynthesis pathway except for CoGA20ox3, CoGA20ox4, CoGA20ox5, and CoGA20ox6. Only one primer was designed for these four genes since the CDS region of these four sequences had almost 80–85% similarity. All the primers are shown in Additional file 1: Table S5. After that, qRT-PCR was performed with PowerUp™SYBER™ Green Master Mix (Thermo Fisher Scientific, USA) by maintaining manufactures protocol. The total reaction mixture was 20 μl and each reaction contained 40 ng of cDNA. It was carried out in a 96 well plate on Quantstudio 5 (Applied Biosystems) with the condition- 50 °C for 2 min, 95 °C for 2 min, 95°Cfor 15 s, 60 °C for 1 min for 45 cycles. For all the qRT-PCR reaction there were three biological replications and three technical replications with negative control (no template). A melting curve was also generated to check primer specificity.

The qRT-PCR data was analyzed with the 2-^ΔΔC^T method [64]. The result was expressed as fold change after normalization with reference genes. Though housekeeping genes are usually used as reference genes for normalization, they may change significantly, depending on the tissue, environmental conditions and species. According to Hossain et al. [32] catalytic subunit of protein phosphatase 2A (*PP2Ac*) gene was used as reference gene for expression analysis in different tissues. However, to observe, the expression changes of GA biosynthesis genes upon GA, PAC and G + P treatment, there was no such study for jute. Therefore, an experiment was conducted to find the housekeeping gene which was not GA regulated (data not shown) and more stable within different treated samples and found glyceraldehyde 3-phosphate dehydrogenase (*GAPDH*) as the desired one.

## Supplementary information

**Additional file 1: Table S1.** Accession numbers of GA metabolism genes used in this paper. **Table S2.** Multilevel consensus sequences for the MEME defined motifs observed among early steps GA biosynthesis genes from rice, *Arabidopsis*, banana and jute. **Table S3.** Multilevel consensus sequences for the MEME defined motifs observed among GA oxidases from rice, *Arabidopsis*, banana and jute. **Table S4.** Activities of *cis*-acting elements in the promoter region of GA biosynthesis genes. **Table S5.** Primer sequences of the reference gene and GA metabolism genes for qRT-PCR in this study.

**Additional file 2: Figure S1.** Multiple alignments of GA20 oxidases from jute. (Green colored box shows GA substrate binding site, brown colored shows 2-oxoglutarate-binding motif (LPWKET), black colored boxes show Fe^2+^-binding motif). **Figure S2.** Multiple alignments of GA3 oxidases from jute. (Black colored boxes show the Fe^2+^-binding motif). **Figure S3.** Multiple alignments of C-19 GA2 oxidases from jute. (Black colored boxes show the Fe^2+^-binding motif). **Figure S4.** Multiple alignments of C-20 GA2 oxidases from jute. (Black colored boxes show the Fe^2+^-binding motif). **Figure S5.***Cis*-acting elements present in the promoter region of GA biosynthetic genes. *Cis*-acting elements responsible for plant hormone regulation (a) for plant growth and development (b) for biotic and abiotic stress response (c) for promoter function (d). 1000 bp upstream sequence from the start point was considered. **Figure S6.** Heat map showing relative expression of genes involved in GA biosynthesis. Samples were taken from 4-day-old seedlings, fiber cells, very young seedlings before bolting and fiber cells. The heat map of normalized RNA-seq data was prepared from three biological replicates from fiber cells and whole seedlings of *C. olitorius* and *C. capsularis*. Gene expression was measured by quantified transcription levels (fragments per kilobase of exon model per million mapped reads, FPKM) derived from RNA-seq analysis. Heat scale, log2 (FPKM). In order to calculate the log2 (FPKM) values of individual genes, all of the original FPKM values were added by a pseudo-count of 1.

## Data Availability

The datasets supporting the conclusions and description of a complete protocol can be found within the manuscript and its additional files. The datasets used and/or analysed during the current study are available from the corresponding author on reasonable request.

## Abbreviations

GA: Gibberellin
CPS: *ent*-copalyl diphosphate synthase
KAO: *ent*-kaurenoic acid oxidase
KO: *ent*-kaurene oxidase
KS: *ent*-kaurene synthase
GA20ox: GA20oxidase
GA2ox: GA2-oxidase
GA3ox: GA3-oxidase
GGDP: Geranylgeranyl diphosphate
TPSs: Terpene synthasese
XET: Xyloglucan endotransglycosylases
NCBI: National Center for Biotechnology Information
qRT-PCR: quantitative Real-Time Polymerase Chain Reaction
PAC: Paclobutrazol
CTAB: Cetyltrimethylammonium bromide
2ODD: 2-oxoglutarate–dependent dioxygenases
PP2Ac: Catalytic subunit of protein phosphatase 2A
GAPDH: Glyceraldehyde 3-phosphate dehydrogenase
TMTT: (E,E)-4,8,12-trimethyltrideca-1,3,7,11-tetraene
