# Supplementary material for: Genome-wide identification, characterization and expression profiling of gibberellin metabolism genes in jute
Source: BMC Plant Biol. 2020 Jul 1;20:306. doi: 10.1186/s12870-020-02512-2 (PMC7329397; doi:10.1186/s12870-020-02512-2)
Supplement: Supplementary file 1 — Additional file 1: Table S1. Accession numbers of GA metabolism genes used in this paper. Table S2. Multilevel consensus sequences for the MEME defined motifs observed among early steps GA biosynthesis genes from rice, Arabidopsis, banana and jute. Table S3. Multilevel consensus sequences for the MEME defined motifs observed among GA oxidases from rice, Arabidopsis, banana and jute. Table S4. Activities of cis-acting elements in the promoter region of GA biosynthesis genes. Table S5. Primer sequences of the reference gene and GA metabolism genes for qRT-PCR in this study. [file 12870_2020_2512_MOESM1_ESM.docx]

**Additional file 1: Table S1.Accession numbers of GA metabolism genes used in this paper.**

| **Enzyme** | **Plant** | **Gene name** | **Accession no.** |
| --- | --- | --- | --- |
| CDP synthase (CPS) | Arabidopsis thaliana | AtCPS | NP_192187 |
|  | *Oryza sativa Japonica Group* | *OsCPS* | BAS78107 |
|  | Musa acuminata | *MaCPS1* | XP_009414733 |
|  |  | *MaCPS2* | XP_009414734 |
|  |  | *MaCPS3* | XP_009415635 |
| *ent*-kaurene synthase (KS) | Arabidopsis thaliana | *AtKS* | NP_178064 |
|  | *Oryza sativa Japonica Group* | *OsKS* | BAS90962 |
|  | Musa acuminata | *MaKS1* | *XP_009381749* |
|  |  | *MaKS2* | XP_009381751 |
| *ent*-kaurene oxidase (KO) | *Arabidopsis thaliana* | *AtKO* | NP_197962 |
|  | *Oryza sativa Japonica Group* | *OsKO* | BAS98310 |
|  | Musa acuminata | *MaKO* | *XP_009403115* |
| *ent*-kaurenoic acid oxidase (KAO) | Arabidopsis thaliana | *AtKAO1* | NP_172008 |
|  |  | *AtKAO2* | NP_001189657 |
|  | *Cucurbita maxima* | *OsKAO* | BAS95781.1 |
|  | Musa acuminata | *MaKAO1* | *XP_009392783* |
|  |  | *MaKAO2* | *XP_009420467* |
| GA 20-oxidase (GA20ox) | Arabidopsis thaliana | *AtGA20ox1* | NP_194272 |
|  |  | *AtGA20ox2* | NP_199994 |
|  |  | *AtGA20ox3* | NP_196337 |
|  |  | *AtGA20ox4* | NP_176294 |
|  |  | *AtGA20ox5* | NP_175075 |
|  | *Oryza sativa Japonica Group* | *OsGA20ox1* | BAF13865 |
|  |  | *OsGA20ox2* | BAJ09478 |
|  |  | *OsGA20ox3* | BAF20901 |
|  |  | *OsGA20ox4* | BAH93154 |
|  |  | *OsGA20ox5* | XP_015628260 |
|  |  | *OsGA20ox6* | XP_015633778 |
|  |  | *OsGA20ox7* | XP_015648463 |
|  |  | *OsGA20ox8* | XP_015635293 |
|  | Musa acuminata | *MaGA20ox1* | *XP_009380434* |
|  |  | *MaGA20ox2* | XP_009396824 |
|  |  | *MaGA20ox3* | XP_009406147 |
|  |  | *MaGA20ox4* | XP_009407673 |
|  |  | *MaGA20ox5* | XP_009407673 |
|  |  | *MaGA20ox6* | XP_009414611 |
|  |  | *MaGA20ox7* | XP_009413747 |
|  |  | *MaGA20ox8* | XP_009383569 |
|  |  | *MaGA20ox9* | XP_009385199 |
|  |  | *MaGA20ox10* | XP_009387900 |
| GA 3-oxidase (GA3ox) | Arabidopsis thaliana | *AtGA3ox1* | NP_173008 |
|  |  | *AtGA3ox2* | NP_178150 |
|  |  | *AtGA3ox3* | NP_193900 |
|  |  | *AtGA3ox4* | NP_178149 |
|  | *Oryza sativa Japonica Group* | *OsGA3ox1* | Q6AT12.1 (uniprot ID) |
|  |  | *OsGA3ox2* | XP_015634638 |
|  | Musa acuminata | *MaGA3ox1* | XP_009390400 |
|  |  | *MaGA3ox2* | XP_009396646 |
|  |  | *MaGA3ox3* | XP_009400517 |
|  |  | *MaGA3ox4* | XP_009409327 |
|  |  | *MaGA3ox5* | XP_009385827 |
| GA 2-oxidase (GA2ox) | Arabidopsis thaliana | *AtGA2ox1* | NP_177965 |
|  |  | *AtGA2ox2* | NP_174296 |
|  |  | *AtGA2ox3* | NP_181002 |
|  |  | *AtGA2ox4* | NP_175233 |
|  |  | *AtGA2ox6* | NP_171742 |
|  |  | *AtGA2ox7* | NP_175509 |
|  |  | *AtGA2ox8* | NP_193852 |
|  | *Oryza sativa Japonica Group* | *OsGA2ox1* | XP_015639483 |
|  |  | *OsGA2ox2* | BAC16751 |
|  |  | *OsGA2ox3* | XP_015649346 |
|  |  | *OsGA2ox4* | AAU03107 |
|  |  | *OsGA2ox5* | XP_015645542 |
|  |  | *OsGA2ox6* | XP_015635159 |
|  |  | *OsGA2ox7* | XP_015633380 |
|  |  | *OsGA2ox8* | XP_015638414 |
|  |  | *OsGA2ox9* | XP_015624176 |
|  |  | *OsGA2ox10* | AAT01379 |
|  | Musa acuminata | *MaGA2ox1* | XP_009394604 |
|  |  | *MaGA2ox2* | XP_009395077 |
|  |  | *MaGA2ox3* | XP_009396510 |
|  |  | *MaGA2ox4* | XP_009405644 |
|  |  | *MaGA2ox5* | XP_009406244 |
|  |  | *MaGA2ox6* | XP_009409401 |
|  |  | *MaGA2ox7* | XP_009412952 |
|  |  | *MaGA2ox8* | XP_009415245 |
|  |  | *MaGA2ox9* | XP_009416515 |
|  |  | *MaGA2ox10* | XP_009417251 |
|  |  | *MaGA2ox11* | XP_009418345 |
|  |  | *MaGA2ox12* | XP_009421396 |
|  |  | *MaGA2ox13* | XP_009380496 |
|  |  | *MaGA2ox14* | XP_009383703 |
|  |  | *MaGA2ox15* | XP_009386085 |

**Additional file 1: Table S2. Multilevel consensus sequences for the MEME deﬁned motifs observed among early steps GA biosynthetic genes from rice, *Arabidopsis*, banana and jute**

| **Motif no** | **width** | **consensus sequence** |
| --- | --- | --- |
| Motif-1 | 50 | HRFGGGVPNVYPVDLFEHLWMVDRLERLGISRYFQQEIKSCLDYVYRYWT |
| Motif-2 | 41 | DWDRIMKYQCKNGSFFFSPSTTAYALMQTGDDNCLCYLQRV |
| Motif-3 | 50 | LHGYDVSAGVFRHFEKDGEFFCFVGQSTQAVTGMYNLNRASQVAFPGEEI |
| Motif-4 | 50 | TRLYIEQYGGSGDVWIGKTLYRMPLVNNDVYLELAKLDYNRCQALHQLEW |
| Motif-5 | 41 | HDRMINTLACVIALKRWTIYPDQCRRGLQFIRENMWRLSDE |
| Motif-6 | 31 | GPQGPCFPQCLQWIVDNQHPDGSWGDHHIFP |
| Motif-7 | 50 | PLVPFRYAHEDTQMNGYLIPKGWKVQIWFYNCHMDPQVWPDPKKWNPSRW |
| Motif-8 | 50 | CPGNDLAKLEISIFLHHFLLGYQLKRSNPKCPVRYLPHPRPTDNCLARIT |
| Motif-9 | 29 | EHMPIGFEIAFPGMLEYAKNLNLEIPYDQ |
| Motif-10 | 29 | DILIMYLNAGHESSGHITMWATVFLQEHP |
| Motif-11 | 50 | PPGDMGWPIIGNMWAFLRAFKSSDPDSFIASFIRRYGRVGIYKAYMFGNP |
| Motif-12 | 50 | KLTFKIIMYIFLSSESEDVMDALEREYTDLNYGVRAMAINIPGFAYHRAL |
| Motif-13 | 49 | RARSFSYNYLREKQAADQVVDKWIITKDLPGEVAYALDFPWYASLPRVE |
| Motif-14 | 50 | CKRVLMDDDHFHPGWPKSTMELIGKKSFVGISYEEHKRLRRLTAAPVNGH |
| Motif-15 | 37 | VKRQKLLYCYFLAAACIFEPERSVERLAWAKTAVMAT |
| Motif-16 | 21 | HMGDGEISISAYDTAWVAMVP |
| Motif-17 | 41 | DDINHHTKQTFLLVAKSFYYAAHCSPAALRSHISEVLFKPV |
| Motif-18 | 50 | DMHKTMAFGAGKRACAGSLQAMLIACAAIGRFVQEFEWRLRDGEEENVDT |
| Motif-19 | 31 | GSVVPRPCKDLFWKMCKILHVFYMKTDGFTS |
| Motif-20 | 50 | DTRPAFQQQLVRHHLQQAWKEWLMAWHSDASDGFGREETGLLLVRTMESC |

**Additional file 1: Table S3. Multilevel consensus sequences for the MEME deﬁned motifs observed among GA oxidases from rice, *Arabidopsis*, banana and jute**

| **Motif no** | **width** | **consensus sequence** |
| --- | --- | --- |
| Motif-1 | 29 | GAFIVNIGDTFQAWTNGRYKSCRHRVVVN |
| Motif-2 | 20 | HTDPQILTILHQDQVGGLQI |
| Motif-3 | 21 | EQIVRACEEWGFFQVVNHGVP |
| Motif-4 | 17 | NDCIMRLNHYPPCPEPD |
| Motif-5 | 15 | YRDFTWGEYKKFTQK |
| Motif-6 | 15 | KERKSMAYFWCPPYD |
| Motif-7 | 21 | EYCEAMKKLACKIMELMAESL |
| Motif-8 | 27 | WGYGSAHIGRFFSKLMWKETFTFRYCP |
| Motif-9 | 29 | KQKAGPATPFGYGNKRIGCNGDMGWVEYL |
| Motif-10 | 11 | DGKWVTVPPDP |
| Motif-11 | 21 | AHECMDRFFALPLCEKQKAQR |
| Motif-12 | 15 | ECEIPVIDLSGPDSG |
| Motif-13 | 15 | HYRADMNTMDAFTKW |
| Motif-14 | 26 | NNSYRWGTPTATSLRQLSWSEAFHIP |
| Motif-15 | 21 | LQQQSNIPKQFIWPDHEKPCF |
| Motif-16 | 21 | VVDYFVNTLGEDFRHMGRVYQ |
| Motif-17 | 21 | MELMQRMEQEQVKFFAQPQCE |
| Motif-18 | 15 | KIAPLPQLMDEGNPR |
| Motif-19 | 15 | DVKKTGYKVGLPRFL |
| Motif-20 | 11 | RLGDNRLGQFE |

**Additional file 1: Table S4.**  Activities of *cis*-acting elements in the promoter region of GA biosynthesis genes.

| Elements | Activity |
| --- | --- |
| TCA-element | cis-acting element involved in salicylic acid responsiveness |
| TGA-element | auxin-responsive element |
| ABRE | cis-acting element involved in the abscisic acid responsiveness |
| TATC-box | cis-acting element involved in gibberellin-responsiveness |
| CGTCA-motif | cis-acting regulatory element involved in the MeJA-responsiveness |
| P-box | gibberellin-responsive element |
| TGACG-motif | cis-acting regulatory element involved in the MeJA-responsiveness |
| SARE | cis-acting element involved in salicylic acid responsiveness |
| GARE-motif | gibberellin-responsive element |
| ATC-motif | part of a conserved DNA module involved in light responsiveness |
| G-box | cis-acting regulatory element involved in light responsiveness |
| GT1-motif | light responsive element |
| ARE | cis-acting regulatory element essential for the anaerobic induction |
| LTR | cis-acting element involved in low-temperature responsiveness |
| TCT-motif | part of a light responsive element |
| WUN-motif | wound-responsive element |
| 3-AF1 binding site | light responsive element |
| Box 4 | part of a conserved DNA module involved in light responsiveness |
| TCCC-motif | part of a light responsive element |
| ATCT-motif | part of a conserved DNA module involved in light responsiveness |
| AE-box | part of a module for light response |
| TC-rich repeats | cis-acting element involved in defense and stress responsiveness |
| CAG-motif | part of a light response element |
| MRE | MYB binding site involved in light responsiveness |
| MBS | MYB binding site involved in drought-inducibility |
| I-box | part of a light responsive element |
| GATA-motif | part of a light responsive element |
| Box II | part of a light responsive element |
| GC motif | enhancer-like element involved in anoxic specific inducibility |
| GA-motif | part of a light responsive element |
| LAMP-element | part of a light responsive element |
| chs-CMA2a | Petroselinum crispum part of a light responsive element |
| AT-rich sequence | element for maximal elicitor-mediated activation (2copies) |
| CAT-box | cis-acting regulatory element related to meristem expression |
| O2-site | cis-acting regulatory element involved in zein metabolism regulation |
| GCN4_motif | cis-regulatory element involved in endosperm expression |
| CAAT-box | common cis-acting element in promoter and enhancer regions |
| TATA-box | core promoter element around -30 of transcription start |

**Additional file 1: Table S5. Primer sequences of the reference gene and GA metabolism genes for qRT-PCR in this study**

| Gene | For ward primer (5'→3') | Reverse primer (3'→5') |
| --- | --- | --- |
| *CoCPS* | GGTTGGGTTCGCATTGAAGA | TTGCAAGCTCAAGGTACACA |
| *CoKS* | CCAATCATCCTCCCAGCTCT | CGGTACCGTGGATTACATGC |
| *CoKO* | CGGCAGGATCGTCTCTATCA | GGGCTGGACTGTACTTCCTA |
| *CoKAO* | TTGGAGCAGGAAGCAGATTG | TAAGGCGCTCGAGCTGATAG |
| *CoGA20ox1* | CAGAGGAAAGCAGGAGAAC | TCTTCACCCATCACGTTC |
| *CoGA20ox2* | TCGATGAGCAAGCTTTCATT | TTTGGCATGGTGGGTAGTAA |
| *CoGA20ox3/ CoGA20ox4/ CoGA20ox5/ CoGA20ox6* | AACCTTGCTTTGATGCCC | AAGCTCTGCCGCTTTTGA |
| *CoGA20ox7* | ACATGGGGACCTTGTTCA | ACGAGTTCGACTGCATGG |
| *CoGA3ox1* | GCCTATAGAGAAACTCCCATCC | CGTCATCGGAGGAAAACTC |
| *CoGA3ox2* | AAAGCTAGCAGGGAGGTTGA | GCTGCTGTAGCCTCATTGAA |
| *CoGA3ox3* | CAACTTTGGCCTCAAGATCA | ATGGGTTAGACCCAATGAGC |
| *CoGA2ox1* | CGGCAGCCTCTATGAGAAG | CGATATGACCTCTTGAGGAACC |
| *CoGA2ox2* | CCATGTCCATACTCTTCCAAG | CCACTCGATGTGTTATGCTC |
| *CoGA2ox3* | GCCCAGATGTTCATGCTT | GGGACTGAAATCCAACTTCC |
| *CoGA2ox4* | TCAAACCCTCCTCTCCTT | GACTGCTTAGACCATGAAGG |
| *CoGA2ox5* | TCCGCTCAGCAGTGAATAAC | AGCTTCAATCCTTCTGCCAT |
| *CoGA2ox6* | CTTCAGCTCTCTCTCCGAGT | CACGGTGGGTATCTGTTGA |
| *CoGA2ox7* | CAGCCTCGACTCTCAGTTCAT | AATTTGGCACGGTGGGTA |
| *CoGA2ox8* | ATGGTGGTGGCTTCACCGAACC | TTCGCACGCCTCCACAATCAG |
| *PP2A* | TTGCTGCTCAGTTCAACCAT | TTGGAGCACTGAAGACGGTA |
| *GAPDH* | GAAGGATCGGTAGGTTGGTG | CCTTGACTTTGAGCTCGTGA |
