# Supplementary material for: Genome-wide identification, characterization and expression profiling of gibberellin metabolism genes in jute
Source: BMC Plant Biol. 2020 Jul 1;20:306. doi: 10.1186/s12870-020-02512-2 (PMC7329397; doi:10.1186/s12870-020-02512-2)
Supplement: Supplementary file 2 — Additional file 2: Figure S1. Multiple alignments of GA20 oxidases from jute. (Green colored box shows GA substrate binding site, brown colored shows 2-oxoglutarate-binding motif (LPWKET), black colored boxes show Fe2+-binding motif). Figure S2. Multiple alignments of GA3 oxidases from jute. (Black colored boxes show the Fe2+-binding motif). Figure S3. Multiple alignments of C-19 GA2 oxidases from jute. (Black colored boxes show the Fe2+-binding motif). Figure S4. Multiple alignments of C-20 GA2 oxidases from jute. (Black colored boxes show the Fe2+-binding motif). Figure S5.Cis-acting elements present in the promoter region of GA biosynthetic genes. Cis-acting elements responsible for plant hormone regulation (a) for plant growth and development (b) for biotic and abiotic stress response (c) for promoter function (d). 1000 bp upstream sequence from the start point was considered. Figure S6. Heat map showing relative expression of genes involved in GA biosynthesis. Samples were taken from 4-day-old seedlings, fiber cells, very young seedlings before bolting and fiber cells. The heat map of normalized RNA-seq data was prepared from three biological replicates from fiber cells and whole seedlings of C. olitorius and C. capsularis. Gene expression was measured by quantified transcription levels (fragments per kilobase of exon model per million mapped reads, FPKM) derived from RNA-seq analysis. Heat scale, log2 (FPKM). In order to calculate the log2 (FPKM) values of individual genes, all of the original FPKM values were added by a pseudo-count of 1. [file 12870_2020_2512_MOESM2_ESM.docx]

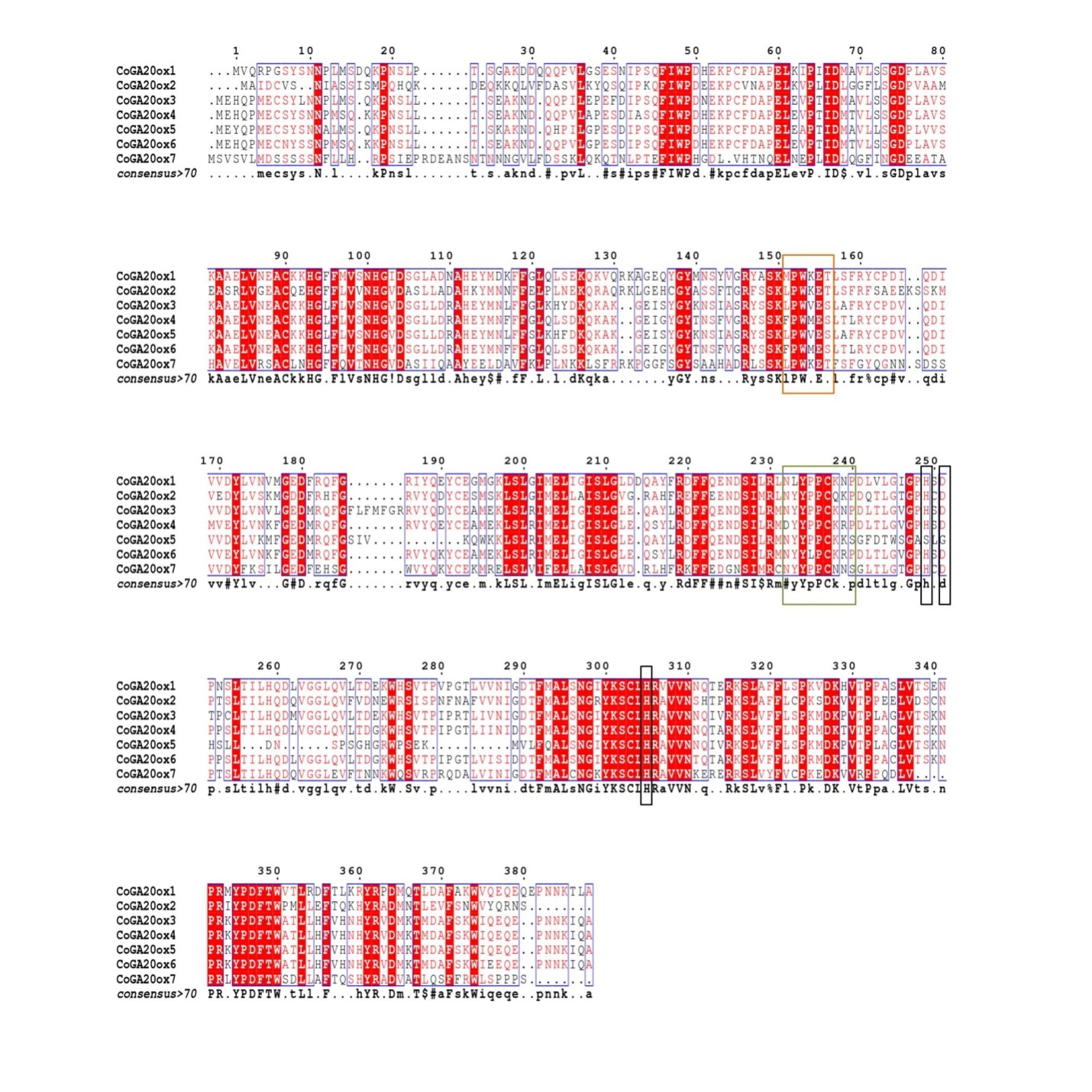


**Additional file 2: Figure S1. Multiple alignments of GA20 oxidases from jute.** (Green colored box shows GA substrate binding site, brown colored shows 2-oxoglutarate-binding motif (LPWKET), black colored boxes show Fe^2+^-binding motif.)


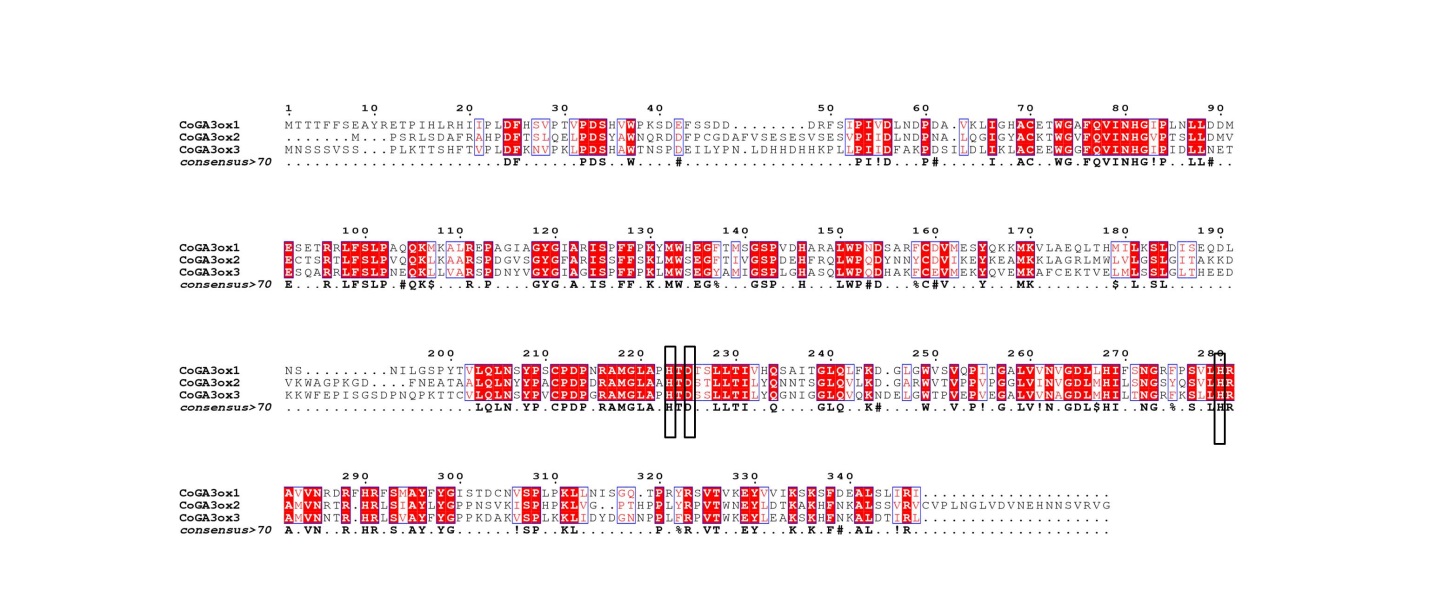


**Additional file 2: Figure S2. Multiple alignments of GA3 oxidases from jute.** (Black colored boxes show the Fe^2+^-binding motif.)


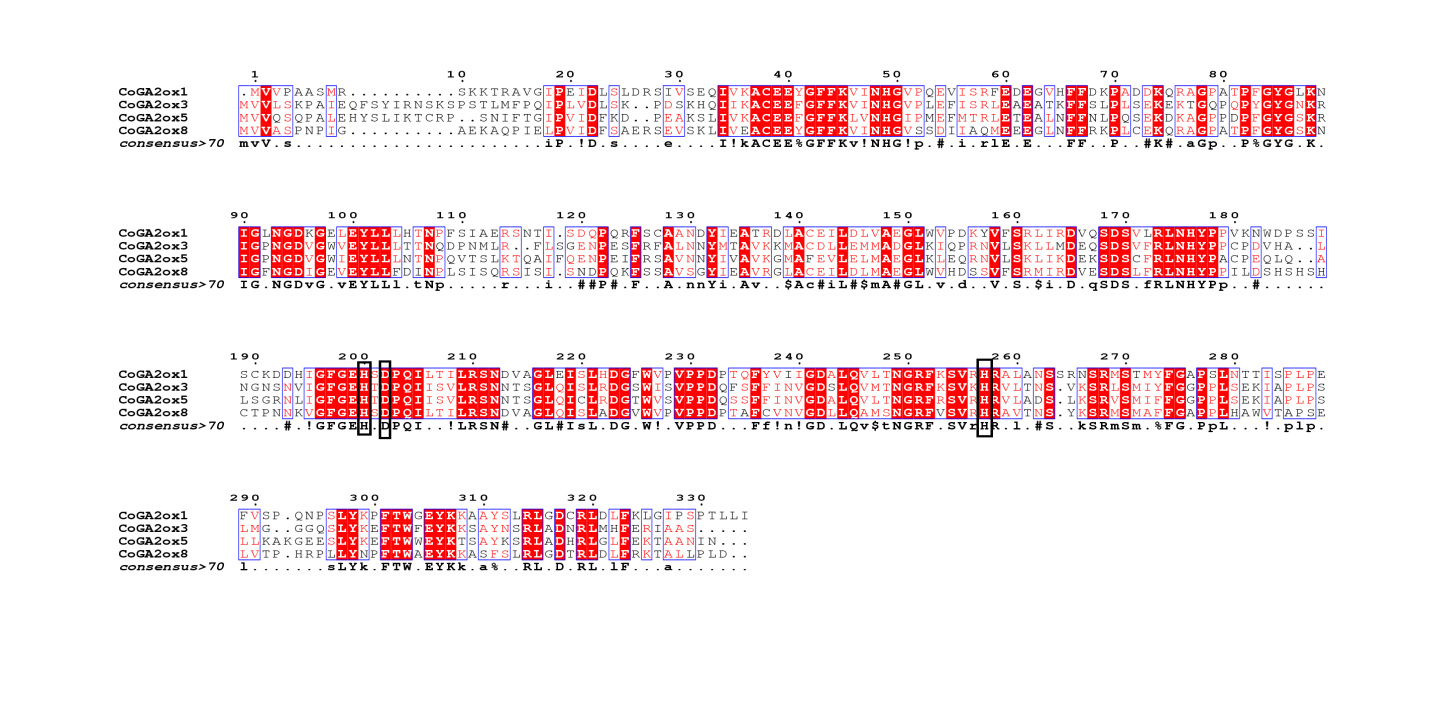


**Additional file 2: Figure S3. Multiple alignments of C-19 GA2oxidases from jute.** (Black colored boxes show the Fe^2+^-binding motif.)


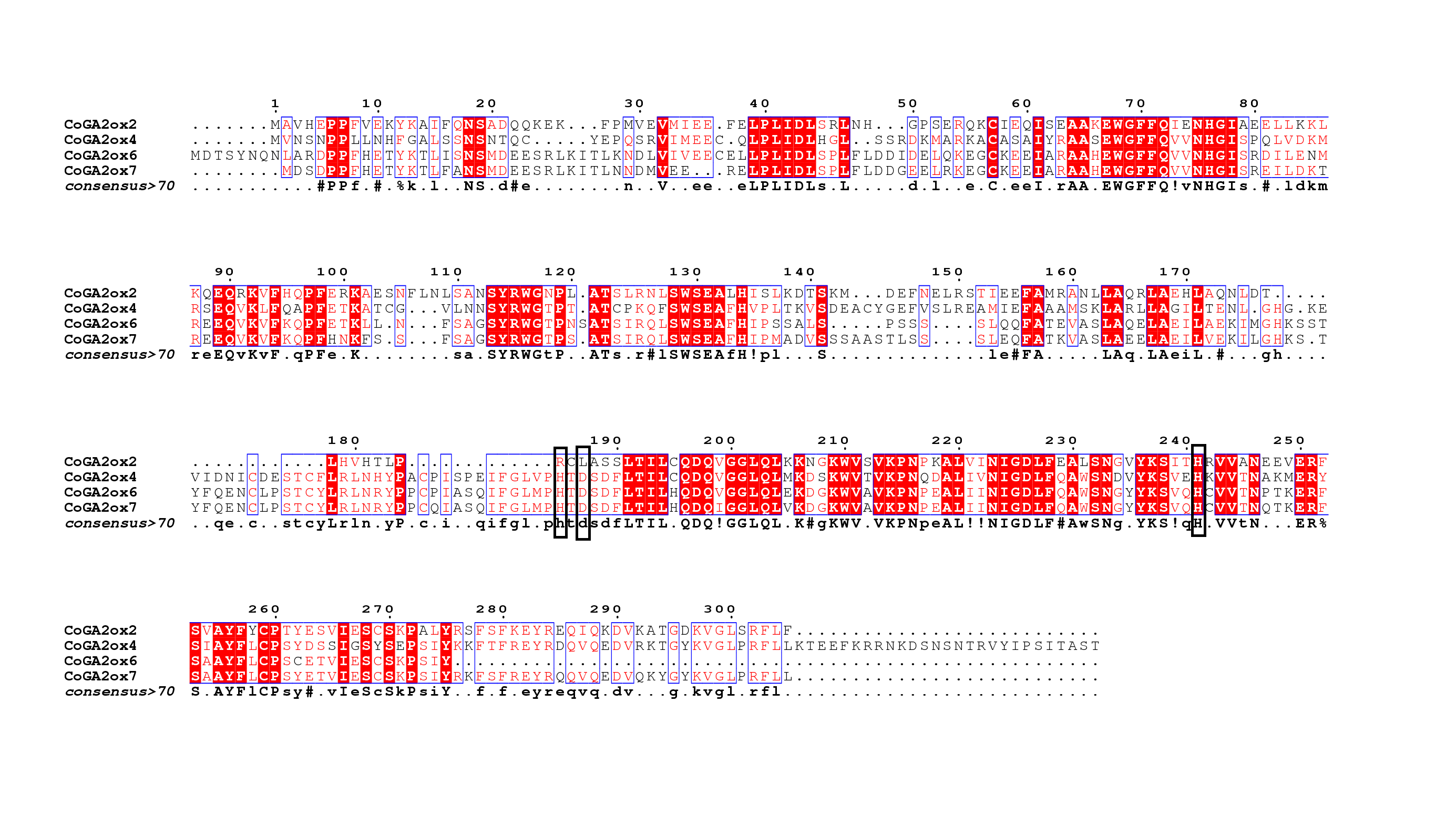


**Additional file 2: Figure S4. Multiple alignments of C-20 GA2oxidases from jute.** (Black colored boxes show the Fe^2+^-binding motif.)


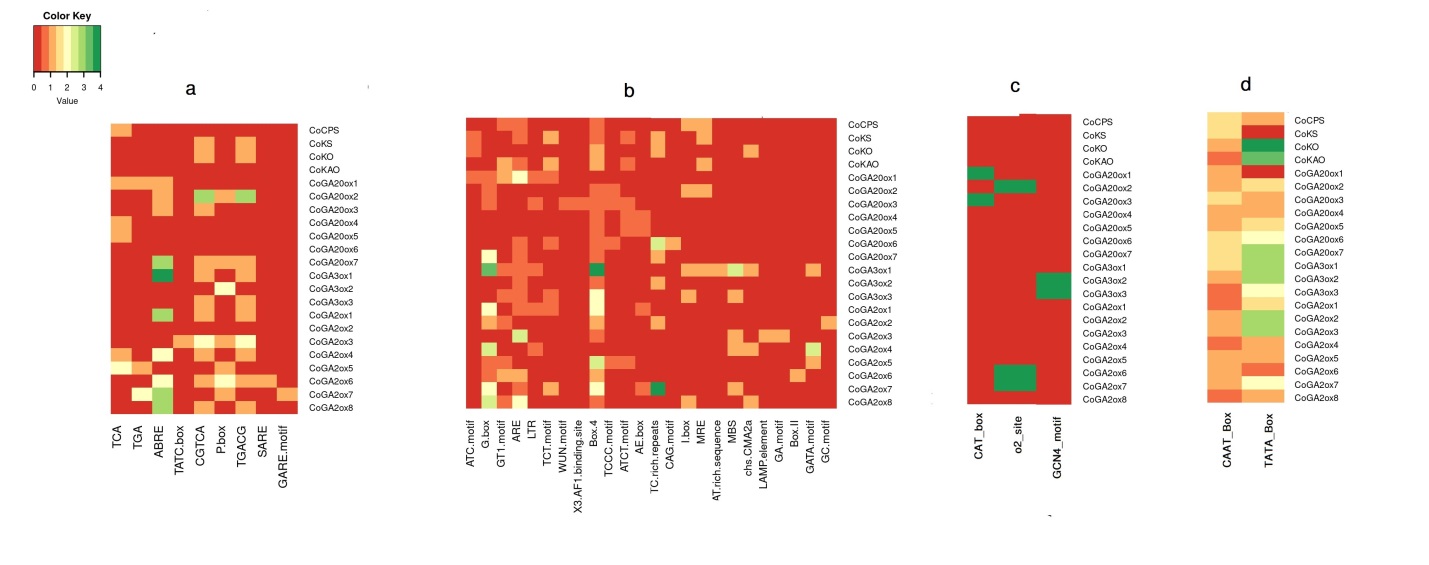


**Additional file 2 Figure S5:** Cis acting elements present in the promoter region of GA biosynthetic genes. *Cis*-acting elements responsible for plant hormone regulation (a) for plant growth and development (b) for biotic and abiotic stress response (c) for promoter function (d). 1000 bp upstream sequence from the start point was considered.


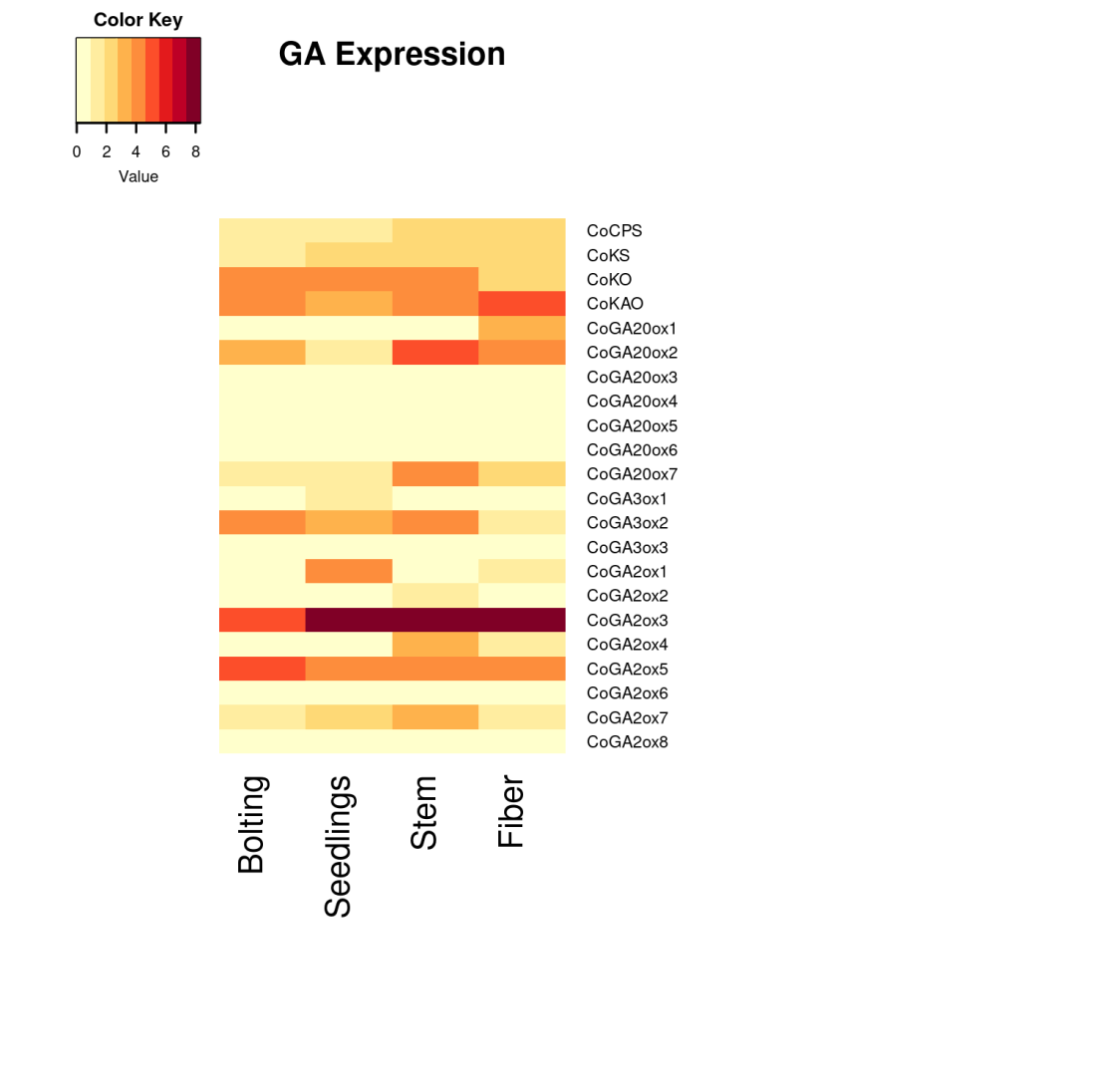


**Additional file 2: Figure S6.** Heat map showing relative expression of genes involved in GA biosynthesis. Samples were taken from 4-day-old seedlings, fibre cells, very young seedlings before bolting and fibre cells. The heat map of normalized RNA-seq data was prepared from three biological replicates from fibre cells and whole seedlings of *C. olitorius* and *C. capsularis*. Gene expression was measured by quantified transcription levels (fragments per kilobase of exon model per million mapped reads, FPKM) derived from RNA-seq analysis. Heat scale, log2 (FPKM). Inorder to calculate the log2 (FPKM) values of individual genes, all of the original FPKM values were added by a pseudo-count of 1.
